# Editorial for Special Issue “Alternatives to Antibiotics: Bacteriocins and Antimicrobial Peptides”

**DOI:** 10.3390/antibiotics11070860

**Published:** 2022-06-27

**Authors:** Joao V. Neves

**Affiliations:** 1 i3S—Instituto de Investigação e Inovação em Saúde, Universidade do Porto, 4150-564 Porto, Portugal; jneves@ibmc.up.pt; 2 Iron and Innate Immunity, IBMC—Instituto de Biologia Celular e Molecular, Universidade do Porto, 4150-564 Porto, Portugal; 3 ICBAS—Instituto de Ciências Biomédicas Abel Salazar, Universidade do Porto, 4150-564 Porto, Portugal

The discovery of penicillin in 1928 by Sir Alexander Fleming, and its later introduction as an antibiotic in the early 1940s, was a gamechanger for the entire medical field [1]. Many medical conditions that up to that time were common causes of death (such as pneumonia, rheumatic fever, gonorrhea and sometimes even a simple scratch that could lead to blood poisoning) became less problematic with the use of this almost miraculous substance with remarkable antimicrobial properties. And so began the Golden Age, during the 1950s–1960s, when almost half of the antibiotics that are still currently in use were discovered. However, soon other problems became apparent, caused by the over-reliance and over-use of antibiotics [2]. Fleming himself had already warned of the possible consequences of their unrestricted use [3], but his words were not heeded, and so we are currently dealing with the disastrous environmental and health-related implications of this abuse. The most concerning problem is the fact that many pathogens are getting more and more resistant to antibiotics, with the emergence of multi-resistant strains that are becoming increasingly problematic for human health, but also in other fields crucial for human survival, such as animal and agricultural production [4]. Additionally, although new, more complex antimicrobial substances with a broader spectrum of activity have been developed in recent years, the odds in this fight are still favoring the pathogens. 

As such, viable alternatives are urgently needed, and antimicrobial peptides (AMPs) are considered some of the best candidates to take up the job [5]. These present several advantages versus common antibiotics: they are highly diverse, being present from the simplest bacteria to the most complex of vertebrates; have a wider range of activity against pathogens, including viruses; have limited resistance development; and usually present more varied methods of action, when compared with the fixed targets of antibiotics. 

This Special Issue includes full research articles and reviews addressing the isolation of novel antimicrobial peptides and bacteriocins, as well as their possible applications and benefits.

Clearly demonstrating the wide array of applications for AMPs, two articles focus on the study of the homofermentative bacterium commonly found in plants and the digestive tract of vertebrates, *Lactiplantibacillus plantarum* (also referred as *Lactobacillus plantarum*), and its antimicrobial roles, with vastly different but equally important objectives. 

Carvalho and collaborators [6] focused on its potential to help prevent or reduce urinary infections derived from the use of urinary tract devices. They compare the proliferation and adhesion of *E. coli* between bare and *L. plantarum* biofilm-covered silicone-based surfaces, clearly showing that *L. plantarum* biofilms present good short-term activity in inhibiting *E. coli* adhesion to the silicone surfaces. They further suggest that the antibiofilm activity provided by *L. plantarum* biofilms can be mainly attributable to the anti-adhesive properties associated with the competitive exclusion mechanism that prevents the adhesion of *E. coli* on the surfaces, and that the killing of *E. coli* may also occur via the production of antimicrobial compounds, such as bacteriocins, hydrogen peroxide, exopolysaccharides, and biosurfactants produced by *L. plantarum*. As such, a *L. plantarum* biofilm coating of silicone-based medical materials has proven to be a promising strategy to avoid the early adhesion and proliferation of pathogens, which may help to reduce urinary tract infections. 

Tenea and Ortega [7] also focused on the study of *L. plantarum*, but with a different approach. Antibiotics are also used in the food industry, from animal farming to healthier crops and food preservation. However, the same risks of their excessive use also apply, often leading to antimicrobial resistance and bioaccumulation in the food chain, so AMPs and bacteriocins can be a viable alternative. In their work, the authors isolated a strain of *L. plantarum* (UTNGt2) from the white cacao plant (*Theobroma grandiflorum*). They show that considering its genetic profile, the ability to produce riboflavin and folate, the absence of acquired antibiotics resistance genes, virulence, and pathogenic factors, and the presence of a diverse array of bacteriocins, it is a nonpathogenic, nonvirulent strain that shows great potential to be further exploited for its probiotic and antimicrobial potential in the food industry or as a potential producer strain of antimicrobial peptides as an alternative to conventional antibiotics. 

On the topic of novel AMPs, Babich and collaborators [8] remind us once again about the enormous diversity of naturally occurring antimicrobial agents and the unusual places where me might find them, by exploring the antimicrobial properties of microorganisms isolated from the bottom sediments of Lake Baikal, located in the mountainous area of Siberia, Russia, north of the border with Mongolia, and considered to be one of the deepest lakes in the world. They isolate various strains of the genera Pseudomonas and Bacillus, as well as some members of the rare genera Micrococcus and Acinetobacter. They further isolate metabolites produced by some of the identified strains and test their antimicrobial properties, concluding that among them are two low-molecular weight oligopeptides, which came to be identified as bacteriocins due to their confirmed antimicrobial activity. The authors state that further studies will have to be performed to ascertain the safety and potential uses of the isolated antimicrobial peptides, but also that this is another step towards a safer, antibiotic restricted future. 

Still focusing on bacteriocins, Velázquez-Suárez and collaborators [9] investigate the antimicrobial activity of the circular bacteriocin AS-48 against *Staphylococcus aureus*, a clinical multidrug-resistant bacterium that can cause various problems, ranging from simple infections to severe sepsis and death. They isolate multiple *S. aureus* strains from different health care units at the University Hospital of Granada in Spain, and investigate the antimicrobial-resistance profile of those isolates, identifying several that are multi-resistant to various antibiotics, more commonly the ones isolated in the ICU and Respiratory and General Pathology services. Since this resistance can pose a serious problem, they then test the effects of AS-48 on those same strains, either alone or in combination with lysozyme. When administered alone, almost all strains showed sensitivity to low concentrations of AS-48, suggestive that its activity is independent of the antimicrobial-resistance profile or genotype. However, together with lysozyme, an antimicrobial peptide that is part of the innate immune system of animals and to which *S. aureus* seems to be naturally resistant, a synergistic effect was observed. Furthermore, the authors also observed that AS-48 was also able to significantly kill cells growing on biofilms, reinforcing the idea that AS-48 can be a viable alternative to treat *S. aureus* infections. 

One of the biggest barriers in the wide-scale use of AMPs is also addressed in this Special Issue by Mirzaee and collaborators [10]: costs of production. There is no denying that AMPs are the future, but large-scale production at a low cost is still not easily achieved. The two most common options for peptide production are recombinant or synthetic peptides, both with pros and cons. Although recombinant production is usually less expensive, low yields, instability and difficulties in purification make it a less than ideal option. However, the costs of synthetic peptides also tend to increase with increased production, complexity and purity. Here, they report on the use of transgenic barley plants to express LL-37, an antimicrobial peptide of the human cathelicidin family with significant pharmaceutical interest. The advantage of barley plants over other vectors to express proteins, such as bacteria, is that they seem to provide a microbial endotoxin-free production, overcoming some of the shortcomings of recombinant production, with the authors also showing that the transgenic barley maintained normal growth through several generations, with the expressed recombinant protein retaining high antibacterial activity. 

Antimicrobial peptides play a pivotal role in invertebrate defenses, as innate immunity components, especially since these organisms lack adaptive immunity. However, much remains to be understood, as these organisms tend to less studied. In this issue, Liu and collaborators [11] focus their attention on lectins in the gazami crab (*Portunus trituberculatus*). Lectins are known to perform an important role in intestinal immunity, and although not considered antimicrobial peptides in the traditional sense, they are nevertheless known to possess strong antimicrobial activities and to induce several immunological factors, including the expression of antimicrobial peptides. Here, they characterize a novel C-type lectin (named PtCLec2) and demonstrate its antimicrobial activity, showing its ability to induce binding and agglutination, as well as triggering the clearance and phagocytosis of the bacterium *Vibrio alginolyticus*. Additionally, they also demonstrate its ability to induce the expression of several immune related genes, including antimicrobial peptides, showing that this lectin might not only function as a mediator for immune recognition (as a pattern recognition receptor, PRR) but also possesses potential immunoregulatory properties, through its antibacterial and opsonic activities.

This Special Issue also includes an extensive review on *Enterococcus* spp. by Almeida-Santos and collaborators [12]. Enterococci are known to be of the most frequent producers of bacteriocins, commonly known as enterocins, giving them an advantage to compete in their natural environment, the human gut and that of many other animals. This review thoroughly documents the current standpoint about the dual role of *Enterococcus* spp. as both producers and targets for bacteriocins and presents an overview of the various enterocins and their spectrum of activity, as well as the challenges of using bacteriocins in the fight against infections, never forgetting the double-edged sword that these represent. On one hand, enterocins are essential for ‘good’ Enterococci to promote a healthy microbiota in the gut, but on the other, they also help ‘bad’ Enterococci to proliferate and cause diseases. As the authors so clearly emphasize, one of the most common causes of hard-to-treat and life-threatening hospital infections is the vancomycin-resistant *E. faecium* which, due to a lack of effective therapeutic options, is recognized by the WHO as a priority pathogen urgently requiring new antimicrobial treatments.

The final contribution to this Special Issue is a review by Amador and collaborators [13], on the subject of lipid transfer proteins (LTPs). These are a class of antimicrobial peptides exclusive to plants, once again showing the huge diversity and potential sources for novel AMPs. Here, they present a thorough review of all aspects of LTPs, from their diversity to functions to possible applications. LTPs represent not only important defenses for plants, presenting a wide spectrum of antimicrobial activity, but might also have other still undetermined roles, suggested by the fact that these peptides are expressed even in the absence of infectious agents. Furthermore, they already have proven benefits for human health, not only in terms of their antimicrobial properties (including antiproliferative activity against viruses such as HIV or H1N1, with SARS-CoV-2 being a good candidate for further studies), but with many more possible applications, such as pharmacological, for the development of new drugs or to provide chemical and physical stability to other compounds, or even in the food industry, to increase protection and shelf life. However, there is still much that is unknown and must be better explored for an effective and safe use of LTPs.

To conclude, this Special Issue collects multidisciplinary research articles that focus on both new and old antimicrobial peptides, further exposing the problems that are derived from the excessive use of antibiotics and reinforcing the need to find alternative solutions. Antimicrobial peptides are some of the strongest candidates for that role, but there is still much to be done in this field until they can successfully complement or even replace antibiotics.
